# Genomic, transcriptomic, and metabolomic analyses provide insights into the evolution and development of a medicinal plant *Saposhnikovia divaricata* (Apiaceae)

**DOI:** 10.1093/hr/uhae105

**Published:** 2024-04-09

**Authors:** Zhen-Hui Wang, Xiao Liu, Yi Cui, Yun-He Wang, Ze-Liang Lv, Lin Cheng, Bao Liu, Hui Liu, Xin-Yang Liu, Michael K Deyholos, Zhong-Ming Han, Li-Min Yang, Ai-Sheng Xiong, Jian Zhang

**Affiliations:** Faculty of Agronomy, Jilin Agricultural University, Changchun 130118, China; College of Chinese Medicinal Materials, Jilin Agricultural University, Changchun 130118, China; College of Chinese Medicinal Materials, Jilin Agricultural University, Changchun 130118, China; College of Chinese Medicinal Materials, Jilin Agricultural University, Changchun 130118, China; College of Chinese Medicinal Materials, Jilin Agricultural University, Changchun 130118, China; College of Chinese Medicinal Materials, Jilin Agricultural University, Changchun 130118, China; Key Laboratory of Molecular Epigenetics of the Ministry of Education, Northeast Normal University, Changchun 130024, China; State Key Laboratory of Crop Genetics & Germplasm Enhancement and Utilization, College of Horticulture, Nanjing Agricultural University, Nanjing 210095, China; Faculty of Agronomy, Jilin Agricultural University, Changchun 130118, China; Department of Biology, University of British Columbia, Okanagan V1V1V7, Canada; College of Chinese Medicinal Materials, Jilin Agricultural University, Changchun 130118, China; College of Chinese Medicinal Materials, Jilin Agricultural University, Changchun 130118, China; State Key Laboratory of Crop Genetics & Germplasm Enhancement and Utilization, College of Horticulture, Nanjing Agricultural University, Nanjing 210095, China; Faculty of Agronomy, Jilin Agricultural University, Changchun 130118, China; Department of Biology, University of British Columbia, Okanagan V1V1V7, Canada

## Abstract

*Saposhnikovia divaricata*, 2n = 2x = 16, as a perennial species, is widely distributed in China, Mongolia, Russia, etc. It is a traditional Chinese herb used to treat tetanus, rubella pruritus, rheumatic arthralgia, and other diseases. Here, we assembled a 2.07 Gb and N50 scaffold length of 227.67 Mb high-quality chromosome-level genome of *S. divaricata* based on the PacBio Sequel II sequencing platform. The total number of genes identified was 42 948, and 42 456 of them were functionally annotated. A total of 85.07% of the genome was composed of repeat sequences, comprised mainly of long terminal repeats (LTRs) which represented 73.7% of the genome sequence. The genome size may have been affected by a recent whole-genome duplication event. Transcriptional and metabolic analyses revealed bolting and non-bolting *S. divaricata* differed in flavonoids, plant hormones, and some pharmacologically active components. The analysis of its genome, transcriptome, and metabolome helped to provide insights into the evolution of bolting and non-bolting phenotypes in wild and cultivated *S. divaricata* and lays the basis for genetic improvement of the species.

## Introduction

*Saposhnikovia divaricata* (Turcz.) Schischk., a perennial herb, has been used for more than 2000 years as medicinal plant in China, Mongolia, Korea, Japan, etc. [1–4]. The dried roots and rhizomes are called ‘Fang Feng’ in China, while they are called ‘Bofu’ in Japan and ‘Bang-Poong’ in Korea [2, 5]. Previous studies revealed that chromones, coumarins, and volatile oils are the main active components of *S. divaricata* [1, 6, 7], and are responsible for its analgesic, anti-cancer, anti-inflammatory, anti-pyretic, anti-convulsant, and anti-coagulant effects [1, 8–11]. Previous studies of *S. divaricata* have mainly focused on its pharmacological effects [9, 12–14], biocontrol potential of rhizospheric fungus [15, 16], physiological and ecological characteristics [17], transcriptomics [18], and antioxidant activity [19]. To date, little information is available about the genetic diversity and evolution of *S. divaricata*.

*Saposhnikovia divaricata* is a member of the Apiaceae family, which encompasses approximately 434 genera and 3780 species [20–22]. The Apiaceae family is known for several vegetable and traditional Chinese herbs including *Petroselinum crispum* (Mill.) Hill, *Daucus carota* L., *Apium graveolens* L., and *Bupleurum chinense* DC., *Conioselinum smithii* (H. Wolff) Pimenov & Kljuykov, *Peucedanum praeruptorum* Dunn, etc. The chromosome-level assembly of the *D. carota* genome in 2016 was a significant milestone in Apiaceae family genomics [23, 24]. This was followed by a draft *A. graveolens* genome assembly, with scaffold N50 35.57 kb [25]. A high-quality genome sequence assembly for coriander [26, 27] and an updated celery genome assembly [28] were reported in 2020 and 2021 by Song *et al.* Recently, T2T (telomere-to-telomere) assemblies have become the ultimate standard for genome sequencing [29–31]. For example, Wang *et al.* (2023) reported a gapless T2T assembly of the carrot variety ‘Kurodagosun’. Based on this, the carotenoid metabolic pathway was reconstructed and the structural genes responsible for regulating carotenoid biosynthesis were identified [32].

High-quality genome sequences assemblies allow comparative analyses of genome architecture and the evolution events [33–35]. Here, we present the initial high-quality genome assembly of *S. divaricata* using combined approaches, including PacBio (PacBio Sequel II) and Hi-C technology. There are eight chromosomes (N50 = 227.67 Mb) in the assembled genome. We annotated repetitive sequences and protein-coding genes and identified expansions in several gene families. The genes, and metabolites relevant to pharmacological activity, flavonoids, and plant hormones were compared between *S. divaricata* bolting and non-bolting types. The new assembly provides a high-quality *S. divaricata* chromosome-level assembly, facilitating the research and crop improvement in this species and others members of the Apiaceae.

## Results

### Genome sequencing and assembly

The genome size, repeat size, and heterozygosity of *S. divaricata* were estimated based on *k*-mer analysis. The 17-mer frequency of Illumina short reads with the highest peak occurred at a depth of 34. The size of genome was estimated to be 1967.45 Mb with 80.55% repeats, and it was estimated that the heterozygosity rate was 0.95% (Table S1 and Fig. S1, see online supplementary material).

**Figure 1:**
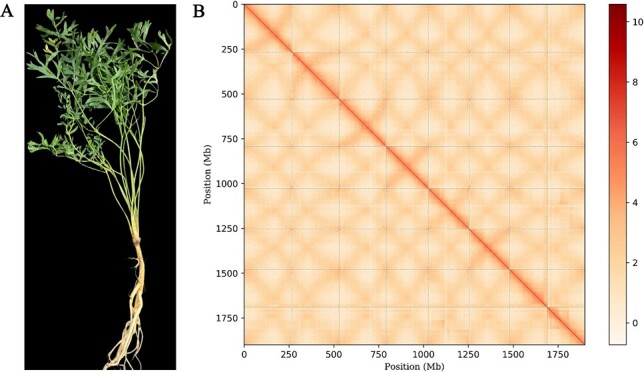
Overview of the *Saposhnikovia divaricata* genome. **A** Morphology of *S. divaricata*. **B** Hi-C heatmap of *S. divaricata* chromosome interactions.

The genome of *S. divaricata* was sequenced and assembled using the Sequel II sequencing platform and contigs of eight pseudochromosomes were anchored and assembled using Hi-C techniques (Fig. 1B).

There were 1899.11 Mb of final chromosome-scale genome in length with 29 contigs, a scaffold N50 = 235.33 Mb, and the maximum pseudochromosome length was 267.13 Mb. The GC content was 35.7% and a contig N50 of 117 Mb (Table 1; Table S2, see online supplementary material). Assessments of the genome quality revealed high gene completeness (BUSCO) [36]. We identified 2193 (98.33%) in the assembly among the 2326 plant-specific orthologs, of which 1400 (97.22%) were considered to be complete (Table S3, see online supplementary material).

**Table 1:** Statistics for the final genome assembly of *Saposhnikovia divaricata*.

Genome information	*S. divaricata* (PacBio + Hi-C)
Sequencing platform	PacBio Sequel
Genome size (Gb)	2.07 Gb
Scaffold number	179
GC content (%)	35.7
N50 length (contig) (Mb)	117 Mb
Predicted Chromosome genes	39 938
Average transcript length (bp)	1613
Average CDS length (bp)	1310
Average exon length (bp)	288
Average intron length (bp)	1061
BUSCO (%)	94.70%

### Repeats and genome annotation

**Figure 2:**
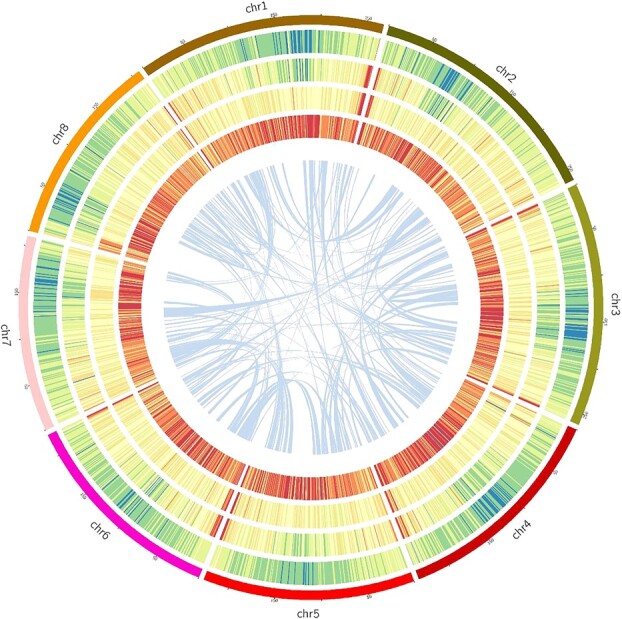
Chromosomal features of *Saposhnikovia divaricata*. Concentric rings from the outside to the inside represent: chromosome position, LTR density, Gypsy density, Copia density, gene density and collinearity. All of these are shown at 1 Mb resolution. The inner lines show syntenic blocks on homologous chromosomes.

By using a combination of *ab initio* and homology-based approaches, 83.47% of the assembled sequences were identified as repetitive sequences, including 73.7% LTR retrotransposons and 9.47% genome DNA transposons (Fig. 2; Table S4, see online supplementary material). A total of 42 984 genes were predicted, of which 39 938 were assigned to chromosomes based on a combination of transcriptome-based, homology-based, and *ab initio* predictions. There were 1631 bp of average transcript length and 1310 bp of coding sequence size, with average exon length was 288 bp (Table 1; Table S5, see online supplementary material). In light of observed gene density, repeat density, and GC content, a circos map of genome of *S. divaricata* was drawn (Fig. 2). Overall, the functions of 42 456 genes (98.77%) were assigned. Among these, 69.34% and 97.96% had predicted homologs in Swiss-Prot and TrEMBL databases, respectively (Table S6, see online supplementary material).

### Comparative genomic analysis

**Figure 3:**
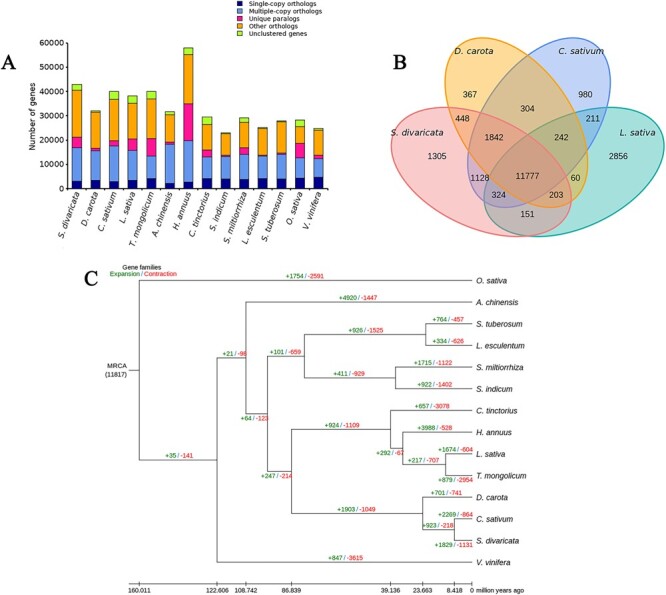
Comparative genomic analysis of the genome of *Saposhnikovia divaricata*. **A** Different species shared the same number of homologous genes. **B** Venn diagram of gene family clustering of *Coriandrum sativum, S. divaricata, Daucus carota*, and *Lactuca sativa*. The numbers represent the number of gene families among *C. sativum, S. divaricata, D. carota*, and *L. sativa.*  **C** Divergence time estimation and gene family expansion/contraction. The numbers marked with red and green represent the contraction and of expansion gene families, respectively.

Fourteen species’ genomes were selected for the purpose of identifying homologous genes, conducting gene family clustering analysis, and examining the enrichment of single copy genes with *S. divaricata* (Fig. 3A). The *S. divaricata* genome consisted of 17 178 gene families, including 992 unique families. The clustering results of the first four species were extracted and plotted in a Venn diagram with the following results (Fig. 3B): a total of 22 198 genes were clustered with *S. divaricata, D. carota, Coriandrum sativum* and *Lactuca sativa* that shared 11 777 genes. *S. divaricata* and *C. sativum* have a close relationship as they shared the most gene families. Additionally, 1305 genes were unique to *S. divaricata*.

### Phylogeny of *S. divaricata*

To estimate the divergence time of the 14 species, we constructed a phylogenetic tree (Fig. 3C). There were 1131 gene family contractions and 1829 gene family expansions detected in *S. divaricata*, which places it most closely to *C. sativum* (864 gene family contractions and 2269 gene family expansions). Furthermore, we found that *C. sativum* and *S. divaricata* separated from their shared ancestor approximately 8.4 million years ago (mya), suggesting a relatively brief divergence time.

Gene family expansion and contraction play a key role in phenotypic adaption during speciation [37–39]. An analysis of KEGG gene family enrichment showed that contracted gene families were mostly related to plant hormone signal transduction, glutathione metabolism and oxidative phosphorylation, while the expanded gene families were involved in protein processing in endoplasmic reticulum, flavonoid biosynthesis and protein export (Figs S2 and S3, see online supplementary material).

### Whole-genome duplications of *S. divaricata*

**Figure 4:**
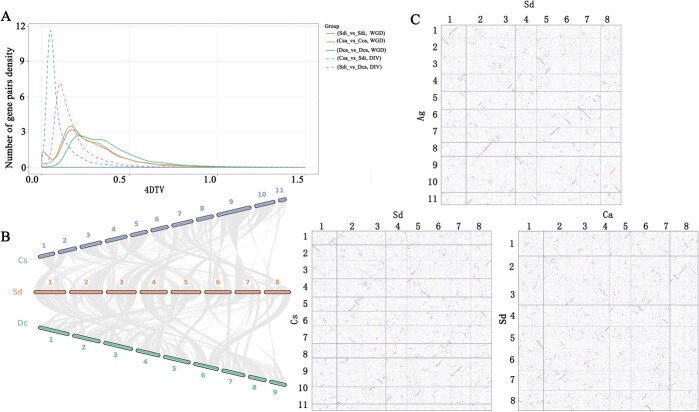
Genome evolution of *Saposhnikovia divaricata* genome. **A**WGD analysis diagram. **B** Collinearity diagram including D. car (*Daucus carota*), Cs (*Coriandrum sativum*) and Sd (*S. divaricata*). **C** Dot plots of Sd vs A. gra (*Apium graveolens* L.) (upper-panel) and Sd vs. Cs (lower-panel).

As can be seen from the density distribution, *C. sativum* shared the smallest peak with *S. divaricata*, and *S. divaricata* had a WGD event near 4DTV ~ 0.176 (Fig. 4A). It was determined that *S. divaricata* experienced one whole-genome duplication (WGD) events based on a synonymous mutation rate analysis of homologous genes.

Analysis of *Ks* values indicated that *D. carota* evolved first, followed by *S. divaricata* and *C. sativum* among these three Apiaceae species. Furthermore, the genomes of *S. divaricata, C. sativum*, and *D. carota* were analysed with an overall syntenic depth ratio of 3:3 by collinearity analysis (Fig. 4B and C), indicating that both species experienced the same WGD events.

### Transcriptome analysis of wild and cultivated *S. divaricata*

**Figure 5:**
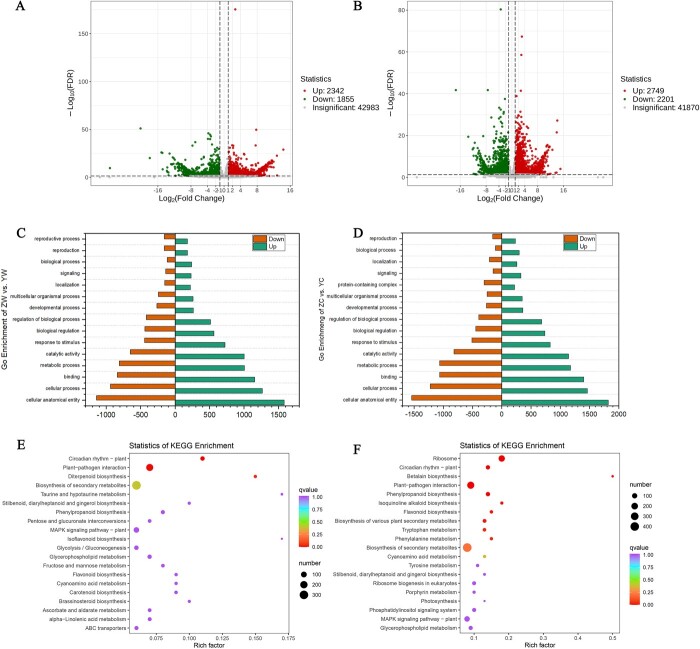
Analysis of transcriptome differences between during wild and cultivated *Saposhnikovia divaricata* in the bolting and non-bolting period. Volcano plots of DEGs (**A**, **B**). DEGs in ZW vs. YW (left, **A**) and ZC vs. YC (right, **B**). The horizontal coordinate shows the log2FoldChange value, and the vertical coordinate shows the –log10 padj or –log10 *P*-value. The threshold line for the differential gene screening criteria is represented by a black dashed line. Upregulated genes are shown in red, and downregulated genes are shown in green (**A**). Cluster dendrogram of differentially expressed genes in wild and cultivated *S. divaricata* without bolting (**B**). GO enrichment with DEGs of wild and cultivated *S. divaricata* with bolting (**C**). GO enrichment with DEGs without bolting (**D**). KEGG enrichment with DEGs with bolting (**E**). KEGG enrichment with DEGs without bolting (**F**). KEGG enrichment with DEGs with bolting. ZW, cultivated *S. divaricata* without bolting; YW, wild *S. divaricata* without bolting; ZC, cultivated *S. divaricata* with bolting; YC, wild *S. divaricata* with bolting.

**Figure 6:**
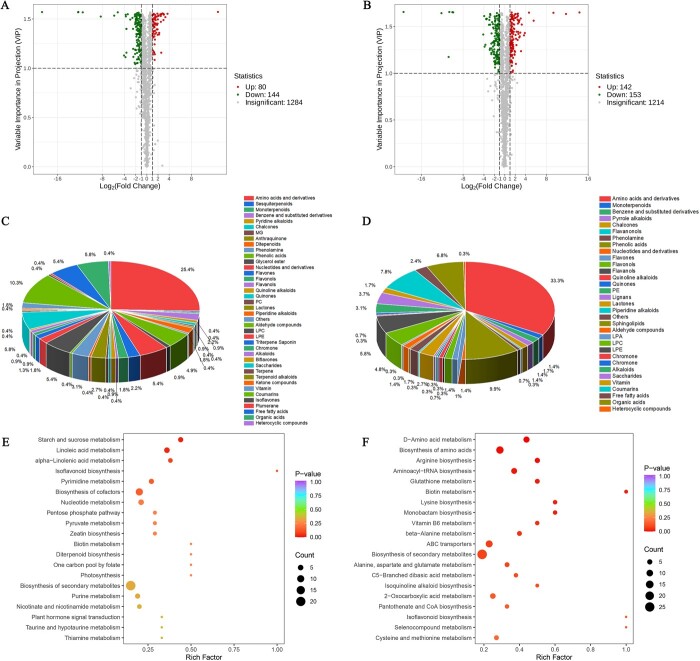
Analysis of effect between bolting and nonbolting during wild and cultivated *Saposhnikovia divaricata* the metabolome. Volcano plots for differentially accumulated metabolites (DAMs) in ZW vs. YW (**A**) and ZC vs. YC (**B**). The horizontal coordinate shows the log2Fold Change value, and the vertical coordinate shows the –log10 padj or –log10 *P*-value. The threshold line for the differential gene screening criteria is represented by black dashed line. Upregulated genes are shown in red and downregulated genes are shown in green (**A** and **B**). Differentially metabolites classes in YW vs ZW (**C**) and differentially metabolites classes in YC vs ZC (**D**). Top KEGG enrichment results. Differential metabolite KEGG enrichment map in YW vs ZW (**E**) and differential metabolite KEGG enrichment map in YC vs ZC (**F**). The horizontal coordinate indicates the Rich Factor corresponding to each pathway, the vertical coordinate is the pathway name (sorted by *P*-value), and the color of the dots reflects the size of the *P*-value, with redder indicating more significant enrichment. The color of the dot reflects the size of the *P*-value, the redder the more significant the enrichment. The size of the dot represents the number of different metabolites enriched. ZW, cultivated *S. divaricata* without bolting; YW, wild *S. divaricata* without bolting; ZC, cultivated *S. divaricata* with bolting; YC, wild *S. divaricata* with bolting.

We studied changes between wild and cultivated *S. divaricata* during the bolting and non-bolting period using transcriptomic and metabolomic analyses. There were 4197 differentially expressed genes (DEGs) identified in the YW vs. ZW comparison (Fig. 5), and there were 2342 genes upregulated and 1855 genes downregulated, respectively (Fig. 5A). Additionally, we found 224 differently abundant, including 144 that decreased and 80 that increased (Fig. 6A), which were annotated with KEGG and GO terms (Fig. 5). According to the comparison of ZC vs. YC, we identified a total of 295 different metabolites and 4950 DEGs, consisting of 2749 upregulated genes and 2201 downregulated genes (Fig. 5B) and 153 decreased and 142 increased metabolites (Fig. 6B), and clustering and annotation of these genes were performed using KEGG and GO terms.

We analysed 15 GO enrichment processes to gain a better understanding of biological processes between wild and cultivated *S. divaricata* during the bolting and non-bolting period. There is a significant enrichment of GO terms of DEGs in ZW vs. YW shown that were ‘cellular anatomical entity’, ‘metabolic process’, ‘catalytic activity’, ‘response to stimulus’, and ‘signaling’ (Fig. 5C). A significant enrichment of GO terms of DEGs in ZC vs. YC (Fig. 5D) were shown to be similar as ZW vs. YW.

According to the KEGG analysis, most of the identified DEGs between ZW vs. YW were enriched in biosynthesis of secondary metabolites (BSM), metabolic pathways and plant hormone signal transduction (PHST, Fig. 5E), while in ZC vs. YC were enriched in BSM, plant−pathogen interaction, PHST and phenylpropanoid biosynthesis (Fig. 5F). Based on the GO and KEGG enrichment results, there were some differences that existed between wild and cultivated *S. divaricata* during the bolting and non-bolting period.

### Metabolome analysis of wild and cultivated *S. divaricata*

There were 1508 metabolites detected in cultivated *S. divaricata* and wild *S. divaricata* without bolting, which 224 different metabolites were identified as differentially abundant. These differentially metabolites belonged to 40 classes, most of them were amino acids and derivatives, terpenoids, flavonoids, and others (Fig. 6C). Similarly, there were 1509 metabolites detected in cultivated *S. divaricata* and wild *S. divaricata* with bolting, which 298 different metabolites, 40 classes were identified, with the majority belonging to amino acids and derivatives, terpenoids, flavonoids, chromone, coumarins, organic acids and others (Fig. 6D).

According to KEGG pathway analysis, the differential metabolites (DMs) were enriched in different pathways. DMs were found in the cultivated *S. divaricata* and wild *S. divaricata* without bolting that were involved in biosynthesis of cofactors, biosynthesis of secondary metabolites, starch and sucrose metabolism and linoleic acid metabolism (Fig. 6E). DMs identified in cultivated *S. divaricata* and wild *S. divaricata* with bolting were involved in biosynthesis of secondary metabolites, amino acid metabolism, ABC transporters and aminoacyl-tRNA biosynthesis (Fig. 6F).

### Analysis of the pharmacological activity components in cultivated and wild *S. divaricata*

The pharmacologically active substances in *S. divaricata* are mainly chromones and coumarins, they are quality indicators of the species [40], chromones are mainly synthesized by flavonoid biosynthesis pathway [2, 8]. Flavonoids are important secondary metabolite, therefore, differentially expressed genes and metabolites in this pathway are significant for the study of the synthesis of chromone. As shown in Table 2, there are significant differences in pharmacological activity ingredients in cultivated and wild *S. divaricata* without bolting. The content of aloesin and 2,5-Dimethyl-7-hydroxychromone belong to chromone, in the cultivated *S. divaricata* were significantly higher than that in wild *S. divaricata*, about 2.2 times as much as that in wild *S. divaricata*. Nevertheless, the content of isofraxetin, phellopterin, coumarin-3-carboxylic acid, rutarin, osthole, praeroside VI, they are belonging to coumarins, in the cultivated *S. divaricata* were significantly less than that in wild *S. divaricata*, in addition to these 17 DMs belonging to coumarins and two DMs belonging to chalcones, were found to be higher in cultivated than in wild species, particularly xanthotoxol and coumestrol, about more than 10 times as much as that in wild *S. divaricata*. At the same time, we identified 12 flavonoid DMs, three up-regulated in the wild species and nine in the cultivated species. The cimifugin concentration did not differ significantly between the two groups. Based on these results, we found that the chromone, coumarins, and flavanols are present in the cultivated and wild *S. divaricata*.

**Table 2:** Analysis of the pharmacological activity components in ZW vs. YW.

Compounds	Class	ZW	YW	VIP	P-value	Log2FC	Type
Aloesin	Chromone	1.85E+06	8.19E+05	1.36E+00	1.20E-02	−1.18E+00	down
2,5-Dimethyl-7-hydroxychromone	Chromone	5.71E+05	2.75E+05	1.12E+00	2.21E-01	-1.06E+00	down
Dalbergin	Coumarins	3.26E+06	8.77E+04	1.57E+00	1.48E-02	−5.22E+00	down
Isofraxetin	Coumarins	2.94E+06	7.02E+06	1.55E+00	2.60E-03	1.26E+00	up
Phellopterin	Coumarins	3.71E+06	8.66E+06	1.42E+00	1.84E-02	1.22E+00	up
Demethylsuberosin	Coumarins	1.13E+07	4.51E+06	1.38E+00	1.14E-01	−1.33E+00	down
Suberosin	Coumarins	1.64E+06	6.54E+05	1.09E+00	2.93E-01	−1.32E+00	down
Alloimperatorin	Coumarins	9.54E+06	2.62E+06	1.51E+00	1.43E-02	−1.86E+00	down
Xanthotoxol; 8-Hydroxypsoralen	Coumarins	9.77E+07	7.16E+06	1.53E+00	1.03E-02	−3.77E+00	down
Daucoidin A	Coumarins	1.99E+05	5.64E+04	1.39E+00	8.16E-02	−1.82E+00	down
Imperatorin	Coumarins	1.28E+07	3.27E+06	1.49E+00	5.11E-04	−1.97E+00	down
Pabulenol	Coumarins	5.43E+05	1.21E+05	1.53E+00	1.99E-02	−2.17E+00	down
Eleutheroside B1	Coumarins	1.43E+05	3.97E+04	1.52E+00	1.02E-02	−1.85E+00	down
Qianhucoumarin G	Coumarins	7.50E+05	2.34E+05	1.50E+00	1.01E-02	−1.68E+00	down
Isoimperatorin	Coumarins	8.83E+06	4.30E+06	1.13E+00	1.97E-01	−1.04E+00	down
Isooxyprohurin	Coumarins	9.13E+04	3.79E+04	1.55E+00	1.28E-02	−1.27E+00	down
7-Hydroxycoumarin;Umbelliferone	Coumarins	2.29E+05	9.88E+04	1.24E+00	5.92E-02	−1.22E+00	down
Coumarin-3-carboxylic Acid	Coumarins	3.77E+04	1.39E+05	1.55E+00	1.16E-02	1.88E+00	up
Rutarin	Coumarins	8.01E+05	3.46E+06	1.53E+00	1.69E-02	2.11E+00	up
Peucedanocoumarin I	Coumarins	1.04E+05	4.64E+04	1.14E+00	1.45E-01	−1.16E+00	down
Osthole	Coumarins	4.21E+05	8.83E+05	1.48E+00	3.21E-02	1.07E+00	up
Hyuganin C	Coumarins	1.25E+05	5.05E+04	1.37E+00	1.17E-02	−1.31E+00	down
Praeroside VI	Coumarins	3.92E+05	1.26E+06	1.52E+00	3.19E-02	1.68E+00	up
Scopoletin-7-O-glucoside (Scopolin)	Coumarins	3.78E+06	1.64E+06	1.13E+00	2.22E-01	−1.21E+00	down
Coumestrol	Coumarins	1.31E+05	1.22E+04	1.55E+00	1.79E-02	−3.42E+00	down
4,4′-Dihydroxy-2′-methoxychalcone (3-Deoxysappanchalcone)	Chalcones	9.12E+06	2.61E+06	1.50E+00	1.11E-02	−1.81E+00	down
Phloretin-4′-O-(6′′-Cinnamoyl)glucoside	Chalcones	3.50E+05	9.14E+04	1.53E+00	6.33E-04	−1.94E+00	down
(+)-Medicarpin	Flavones	3.35E+05	1.05E+05	1.44E+00	4.63E-02	−1.67E+00	down
5-Hydroxy-3,7,3′,4′-tetramethoxyflavone (Retusin)	Flavones	1.35E+05	5.19E+04	1.44E+00	2.81E-02	−1.38E+00	down
2-Methyl-5,7,8-trimethoxyisoflavone	Flavones	1.40E+07	3.99E+06	1.49E+00	7.23E-02	−1.82E+00	down
5,2′-Dihydroxy-7,8-dimethoxyflavone glycoside	Flavones	3.03E+05	5.02E+04	1.51E+00	4.16E-02	−2.59E+00	down
Apigenin-6,8-di-C-arabinoside^*^	Flavones	4.61E+04	9.00E+00	1.57E+00	7.89E-02	−1.23E+01	down
Kaempferol-3-O-galactoside (Trifolin)	Flavonols	3.84E+05	8.30E+05	1.47E+00	4.26E-03	1.11E+00	up
Quercetin-7-O-glucoside	Flavonols	1.45E+05	3.24E+05	1.54E+00	1.74E-02	1.16E+00	up
Kaempferol-3-O-(2′′-O-acetyl)glucuronide	Flavonols	5.92E+05	1.50E+05	1.48E+00	1.10E-03	−1.98E+00	down
Kaempferol-3-O-sophoroside	Flavonols	3.68E+04	7.59E+04	1.50E+00	2.66E-02	1.04E+00	up
Catechin-5-O-glucoside	Flavanols	1.23E+05	4.01E+04	1.38E+00	1.05E-01	−1.61E+00	down
Sciadopitysin	Biflavones	4.68E+06	2.28E+06	1.06E+00	1.92E-01	−1.04E+00	down
7-hydroxy-3-(2-methoxyphenyl)-4H-chromen-4-one	Isoflavones	2.28E+07	3.93E+05	1.53E+00	7.94E-02	−5.85E+00	down

The content of aloesin and 5,7-dihydroxy-2-methylchromone (which are chromones), were significantly higher in cultivated as compared to wild *S. divaricata* with bolting, about 2.6 and 7.1 times as much as that in wild *S. divaricata*, separately. However, the content of dicumarol, coumarin-3-carboxylic Acid, rutarin and praeroside VI, which are coumarins, in the cultivated *S. divaricata* were significantly less than in wild *S. divaricata*. In addition, 19 other metabolites were found to be more abundant in cultivated than in wild plants, particularly fraxetin-8-O-glucoside (fraxin), which was about 707 731 higher in cultivated than in wild *S. divaricata*. At the same time, we identified seven flavonoid DMs, of which two were up-regulated in the wild species and five in the cultivated species. Based on these results, we found that there were differences among chromone, coumarins, and flavanols between cultivated and wild *S. divaricata*. Therefore, we conclude that differences in the accumulation of active ingredients between cultivated and wild *S. divaricata* provide insight into the different properties of these species.

## Discussion

*S. divaricata* is a medicinal herb widely used in clinical practice. To better understand it pharmacological properties as well as the genetics and evolution of species within Apiaceae, we sequenced the genome of *S. divaricata,* provided resources of a high-quality chromosome-level *S. divaricata* reference genome and transcriptome for both fundamental and applied research into Apiaceae plants. The first chromosome-level genome data for *S. divaricata* were reported here. The genome size of *S. divaricata* was estimated to be 2.07 Gb, which is far bigger than that of *D. carota*, closer to the genome sizes of *A. graveolens* and *C. sativum* [27, 28].

The *S. divaricata* genome is mainly composed of repetitive sequences, which account for 85.07% of the genome, which is 1.85 times greater than carrot (45.95%), 1.21 times greater than coriander (70.59%), and 1.24 times greater than celery (68.88%). We found that the percentage of repetitive sequences was significantly greater than that of other species by comparison of genome compositions of species with different genome sizes [26, 28]. For angiosperms, WGD events and tandem duplications are the most important factors determining genome size variation [41, 42]. This genome duplication event not only led to the expansion/contraction of the *S. divaricata* genome, but also may have contributed to the physiological and morphological diversity of the *S. divaricata* lineage.

As a medicinal herb, there are a number of pharmacologically active components from *S. divaricata* and the biosynthesis and metabolism of these compounds are a major area of research. These newly sequenced genomes data provide new views into the unique biosynthetic processes of *S. divaricata*, particularly for these important secondary metabolite pathways. Chromones are the main chemical constituents of *S. divaricata*, and are the main effective components in its antipyretic activity. It has been demonstrated that 4’-O-b-D-glucosyl-5-O-methylvisamminol in *S. divaricata* has significant effects [43, 44]. Using metabolomics, we observed that both 5-O-methylvisamminol and cimifugin are present in both in wild and cultivated *S. divaricata*, and the difference in their abundance is not statistically significant. Our studies on the cultivated and wild *S. divaricata* chromones indicate the presence of aloesin and noreugenin; 5,7-Dihydroxy-2-Methylchromone content in cultivated *S. divaricata* were increased, most of the coumarins and flavonoids content were shown to increase. The contents of the two chromones were significantly higher before bolting comparing to after, indicating that the medicinal quality of *S. divaricata* significantly decreased after bolting (Tables 2 and 3).

**Table 3:** Analysis of the pharmacological activity components in ZC vs. YC.

Compounds	Class	ZC	YC	VIP	P-value	Log2FC	Type
Aloesin	Chromone	1 258 217	468151.6	1.50E+00	2.91E-02	−1.43E+00	down
Noreugenin; 5,7-Dihydroxy−2-Methylchromone	Chromone	700665.5	98254.39	1.27E+00	3.13E-01	-2.83E+00	down
(R)-Columbianetin	Coumarins	9 586 559	3 349 586	1.18E+00	2.66E-01	−1.52E+00	down
(S)-Columbianetin	Coumarins	10 459 816	3 627 814	1.21E+00	2.60E-01	−1.53E+00	down
5,7-Dimethoxycoumarin (Limettin)(Citropten)	Coumarins	346370.4	137764.2	1.25E+00	2.29E-01	−1.33E+00	down
Bakuchicin	Coumarins	43 780 197	7 526 066	1.43E+00	2.14E-01	−2.54E+00	down
Marmesin	Coumarins	9 899 543	3 239 463	1.19E+00	2.69E-01	−1.61E+00	down
Osthenol	Coumarins	64 544 533	3 681 671	1.41E+00	2.15E-01	−4.13E+00	down
Decursinol	Coumarins	6 642 603	2 401 208	1.18E+00	2.69E-01	−1.47E+00	down
Rutaretin	Coumarins	458896.2	170329.4	1.39E+00	1.37E-01	−1.43E+00	down
Fraxetin-8-O-glucoside (Fraxin)	Coumarins	6 369 587	9	1.66E+00	7.31E-02	−1.94E+01	down
Alloimperatorin	Coumarins	27 196 122	2 296 227	1.58E+00	1.38E-01	−3.57E+00	down
Imperatorin^*^	Coumarins	37 498 397	3 422 328	1.58E+00	1.28E-01	−3.45E+00	down
Pabulenol	Coumarins	779900.9	94964.96	1.52E+00	1.54E-01	−3.04E+00	down
Dicumarol	Coumarins	54989.38	146514.1	1.11E+00	3.07E-01	1.41E+00	up
Qianhucoumarin G	Coumarins	2 107 731	280300.7	1.60E+00	7.00E-02	−2.91E+00	down
Dihydroseselin	Coumarins	2 481 295	1 062 942	1.36E+00	2.35E-02	−1.22E+00	down
Isooxyprohurin	Coumarins	226 308	57312.64	1.34E+00	1.43E-01	−1.98E+00	down
Nodakenin	Coumarins	2 926 249	1 012 194	1.15E+00	2.42E-01	−1.53E+00	down
7-Hydroxycoumarin; Umbelliferone	Coumarins	1 424 543	236296.4	1.32E+00	1.59E-01	−2.59E+00	down
Coumarin-3-carboxylic Acid	Coumarins	45986.72	188618.4	1.63E+00	1.53E-02	2.04E+00	up
Rutarin	Coumarins	1 252 843	4 398 364	1.62E+00	2.22E-04	1.81E+00	up
Neobyakangelicol	Coumarins	80141.85	39897.16	1.09E+00	2.42E-01	−1.01E+00	down
Praeroside VI	Coumarins	431 043	2 322 590	1.63E+00	2.40E-02	2.43E+00	up
Coumestrol	Coumarins	44728.24	13640.23	1.48E+00	1.16E-01	−1.71E+00	down
(+)-Medicarpin	Flavones	613 772	144162.2	1.34E+00	2.02E-01	−2.09E+00	down
2-Methyl-5,7,8-trimethoxyisoflavone	Flavones	30 186 068	6 270 526	1.53E+00	3.28E-02	−2.27E+00	down
Luteolin-3’-O-glucoside^*^	Flavones	263617.3	121714.2	1.52E+00	4.95E-02	−1.11E+00	down
Kaempferol-3-O-galactoside (Trifolin)	Flavonols	288600.9	597648.2	1.37E+00	4.30E-02	1.05E+00	up
Kaempferol-3-O-(2′′-O-acetyl)glucuronide	Flavonols	312176.1	128470.6	1.17E+00	1.54E-01	−1.28E+00	down
Kaempferol-7-O-glucoside	Flavonols	336431.3	701196.6	1.57E+00	8.21E-03	1.06E+00	up
Quercetin-3-O-glucoside (Isoquercitrin)	Flavonols	304548.6	150090.5	1.35E+00	3.16E-02	−1.02E+00	down

The composition of essential oils from *S. divaricata* is more complex than other bioactive constituents, being composed of both terpenoids and aliphatic compounds. There is a lack of knowledge about the pharmacological activity of essential oils and their constituents. We analysed terpenoids and aliphatic compounds and found differences between cultivated and wild *S. divaricata* (Tables 2 and 3).

As the main component of Chinese herbal medicines, plant-based Chinese herbal medicines can be divided into wild products and cultivated species according to their different growth modes. The variation in the quality of *S. divaricata* samples from various sources were different because of the different environmental conditions and cultivated technology. Gao *et al.* (2023) observed that wild *S. divaricata* had better quality than annual cultivated products, and perennial *S. divaricata* had better quality than one-year-old *S. divaricata* [40]. Previous reports have shown that a substantial loss of medicinal nutrients after bolting results in a significant decline in the quality of *S. divaricata* [18]. The bolting *S. divaricata* is not included in Pharmacopoeia of the People's Republic of China (PPRC). The results showed that most of the pharmacological activity components from cultivated varieties were higher than those of wild varieties both before and after bolting. We speculate that this may be due to the long-term cultivation of varieties; this study used varieties that have been cultivated for three years, and wild varieties of unknown years. These factors should be considered in future studies and validations.

## Conclusions

We reported the first high-quality chromosome-level genome of *S. divaricata*. The genome size is ∼2.07 Gb, with a contig N50 of 117 Mb. Approximately 99% of the assembled sequences were represented by eight pseudochromosomes. About 85.07 of the repetitive sequences and 39 938 protein-coding genes were identified. The *S. divaricata* genome experienced one recent WGD event, at ∼8.418 Mya. Transcription and metabolism analysis revealed differences in pharmacologically active components, flavonoids, and plant hormones related to plant hormones between wild and cultivated *S. divaricata* before and after bolting. This research will be valuable for further studies of pharmacological activity with cultivated and wild *S. divaricata*.

## Materials and methods

### Plant materials

#### Sample collection and extraction of DNA, RNA, and metabolites

*S. divaricata* was obtained from Jilin Agricultural University, China. Fresh leaves were used for isolation of high molecular weight genomic DNA using the CTAB method. Ten bolting and ten non-bolting cultivated *S. divaricata* plants were collected from Yongmao Farm, Baicheng City, Jilin Province. Ten bolting and ten non-bolting wild *S. divaricata* were collected from Qingshan meadow, Baicheng City, Jilin Province. The first 10 cm from the root-shoot junction of the whole root of each was used for the preparation of LC–MS samples. Total RNA was extracted from the first 10 cm length of the whole root using the Trizol method.

#### Genome sequencing, assembly, and gene annotation

The PacBio Sequel II was used to sequence SMRTbell libraries and ccs software (https://github.com/pacificbiosciences/unanimity) with the parameter ‘-minPasses 3’ used to generate consensus reads (HiFi reads). These long (~15 kb) and highly accurate (>99%) HiFi reads were assembled using hifiasm v. 0.14-r312 [45] with default parameters, and the gfatools (https://github.com/lh3/gfatools) was used to convert sequence graphs in the GFA to FASTA format. A combination of *de novo* gene predictions, homologous gene identification, and alignment to unigene clusters was used to define the gene model. LTR-FINDER [46] and RepeatScout [47] was used to identify repeat elements and RepeatMasker [48] was used to annotate them with the parameter ‘-nolow -no_is -norna -engine wublast’.

#### Hi-C sequencing

The Hi-C library was constructed using digested genomic DNA and it was then sequenced using the Illumina Novaseq platform, Illumina (San Diego, California, USA). The Hi-C Sequencing anchored the scaffolds on eight chromosomes of *S. divaricata* mainly performed with references [49, 50].

#### Gene family cluster analysis

The proteins of the longest transcripts of each gene of *S. divaricata* and closely related species *D. carota*, *C.* sativum, and *L. sativa* were clustered (*S. divaricata, D. carota, C. sativum, L. sativa, T. mongolicum, A. chinensis*). Each gene was filtered for alternative splicing products and only the longest transcripts were retained as coding regions. A protein-coding gene with an ostensibly complete CDS is defined as having a CDS that begins at codon boundary with a start codon, ends at a codon boundary with a stop codon, and has no internal stop codons. Gene family construction was performed using OrthoFinder2 (https://github.com/davidemms/OrthoFinder) using protein sequences from these six species. GO and KEGG analyses were conducted using ClusterProfiler.

### Phylogenetic tree construction

The phylogenetic tree of *S. divaricata* and closely related species was constructed using protein sequences from 412 single-copy ortholog genes. These sequences were aligned using MUSCLE (v3.8.31) [51], and the corresponding coding DNA sequences (CDS) alignments were generated and concatenated accordingly. RAxML (v8.2.11) [52] was used to construct the phylogenetic tree with the maximum likelihood method.

### Gene family contraction and expansion

The gene family expansions and contractions were identified using CAFE [53]. A random birth and death model was used to calculate conditional *P*-values for each gene family across a specified phylogenetic tree. Families with conditional *P*-values less than 0.05 were considered to have an accelerated rate of gene gain or loss and these families were mapped to KEGG pathways for functional enrichment analysis. Hypergeometric tests were used to calculate q-values (FDR, false discovery rate) using the qvalue R package (https://github.com/StoreyLab/qvalue).

### Collinearity analysis

Genome collinearity between the *S. divaricata* and closely related species was performed using MCScanX [54]. Interspecific homologous genes were characterized using BLASTP with the default parameters.

### Whole-genome duplication analysis

We conducted all-versus-all blastp (e-value <1e-5) alignments to detect orthologous genes. Syntenic paralogous blocks and the paralogous and orthologous gene pairs from syntenic blocks were identified and extracted using MCScanX and the HKY substitution model. The 4DTV values and rate of transversions on 4-fold degenerate synonymous sites were further calculated. Then, the potential WGD events were evaluated in each genome based on their 4DTV distribution.

### Transcriptome analysis

We used bowtie2 [55] to map all clean RNA-seq reads to our assembled *S. divaricata* reference genome. The FPKM of each gene was calculated using RNS-Seq by Expectation Maximization (RESM; http://deweylab.github.io/RSEM/). DESeq2 [56] was used to identify treatments that were differentially expressed between treatments, with a FDR<0.05，and log2FC(fold change)>1 or < − 1.

### Metabolome analysis

LC–MS raw data files were converted into mzXML format by ProteoWizard software. We discarded any peaks that had a detection rate lower than 50% in any group of samples. Metabolite identities were assigned using a custom database integrated with public databases, an AI database, and metDNA. Variable importance of projection (VIP) values were calculated using OPLS-DA in the R package MetaboAnalystR, which also reported score plots and permutation plots. Metabolites identification was performed using the KEGG compound database. Annotated metabolites were then mapped to the KEGG Pathway database. Significantly enriched pathways were identified using the *P*-value from a hypergeometric test for a set of metabolites.

## Acknowledgements

This research was supported by a Chinese Key Research and Development Project grant (2019YFC1710700) awarded to L.-M.Y., Promotion Demonstration Project of Forestry Science and Technology of China (JLT2024–30) awarded to Z.-M.H., Jilin Agricultural University high level researcher grant (JLAUHLRG20102006) awarded to J.Z.

## Author contributions

Z.-M.H., A.-S.X., and J.Z. conceived the project. Z.-M.H., Y.C., and X.-Y.L. designed the experiments. Z.-H.W., Y.C., X.L., Y.-H.W., Z.-L.L, L.-M. Y., and H.L. performed most of the experiments. Z.-H.W., Y.C., X.L., Y.-H.W., L.C., B.L., Z.-M.H., and J.Z. analysed and discussed the data. Z.-H.W., A.-S.X., M.K.D., and J.Z. wrote the manuscript with comments from all authors.

## Data availability

Raw sequencing reads have been deposited in the National Center for Biotechnology Information under BioProject accession number PRJNA1041486 and National Genomics Data Center under the project number PRJCA022150.

## Conflict of interest statement

The authors report no conflict of interest.

## Supplementary data

Supplementary data is available at *Horticulture Research* online.

## Supplementary Material

Web_Material_uhae105
