# Supplementary material for: Genomic, transcriptomic, and metabolomic analyses provide insights into the evolution and development of a medicinal plant Saposhnikovia divaricata (Apiaceae)
Source: Hortic Res. 2024 Apr 9;11(6):uhae105. doi: 10.1093/hr/uhae105 (PMC11179723; doi:10.1093/hr/uhae105)
Supplement: Web_Material_uhae105 [file web_material_uhae105.zip › 20240306 Supplementary_files R1.docx]

Fig.S1 K-mer Depth and frequency distribution of *K*-mer


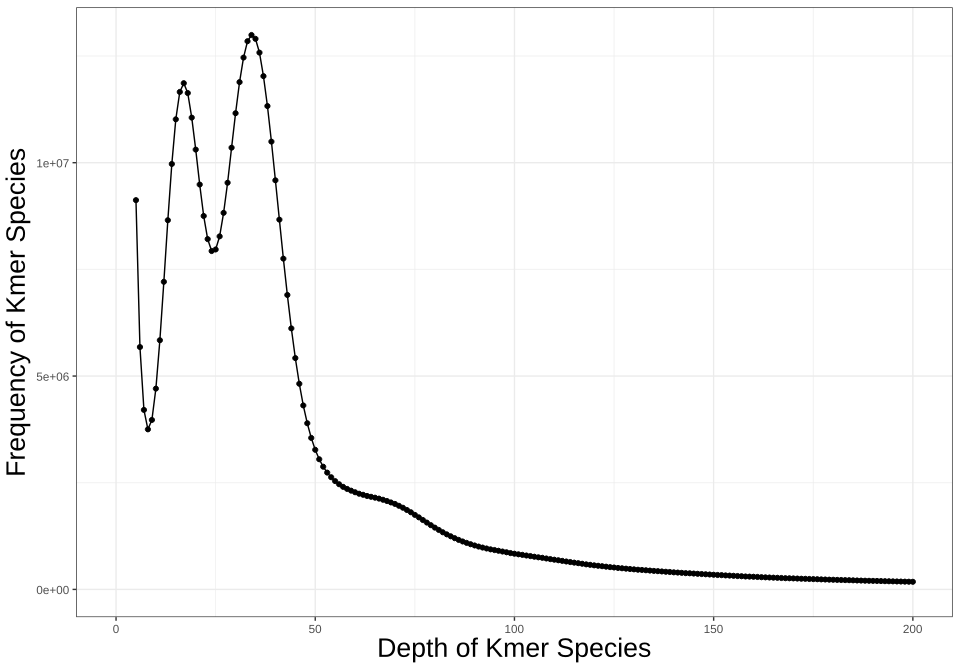


Fig. S2 KEGG enrichment of contraction gene family

Fig.S3 KEGG enrichment of expansion gene family

| Supplementary Table 1 Statistics of Genome characteristics | | | | | |
| --- | --- | --- | --- | --- | --- |
| Sample | K-mer number | K-mer Depth | Genome Size(Mb) | Heterozygous Ratio (%) | Repeat (%) |
| Saposhnikovia_divaricata | 6.89E+10 | 34 | 1967.45 | 0.95 | 80.55 |

| Supplementary Table 2 Statistics for the final genome assembly by Hi-C | | | |
| --- | --- | --- | --- |
| Superscaffold | Number of Contigs | Length of Contigs | Length of Superscaffold |
| Superscaffold1 | 3 | 267,129,953 | 267,130,953 |
| Superscaffold2 | 6 | 262,481,964 | 262,484,464 |
| Superscaffold3 | 5 | 262,084,617 | 262,086,617 |
| Superscaffold4 | 3 | 235,326,616 | 235,327,616 |
| Superscaffold5 | 2 | 227,670,068 | 227,670,568 |
| Superscaffold6 | 3 | 224,685,241 | 224,686,241 |
| Superscaffold7 | 2 | 210,861,088 | 210,861,588 |
| Superscaffold8 | 5 | 208,872,438 | 208,874,438 |
| TOTAL | 29 | 1,899,111,985 | 1,899,122,485 |

| Supplementary Table 3 The Results of BUSCO | | |
| --- | --- | --- |
| Term | BUSCO number | Proportion(%) |
| Complete BUSCOs | 2,193 | 94.3 |
| Complete and single-copy BUSCOs | 2,018 | 86.8 |
| Complete and duplicated BUSCOs | 175 | 7.5 |
| Fragmented BUSCOs | 19 | 0.8 |
| Missing BUSCOs | 114 | 4.9 |
| Total BUSCO groups searched | 2,326 | 100 |

| Supplementary Table 4 Statistics of Repeat sequence classification | | | | | | | | |
| --- | --- | --- | --- | --- | --- | --- | --- | --- |
| Type | RepeatMasker TEs Length (Bp) | RepeatMasker TEs % in genome | RepeatProteinMask TEs Length (Bp) | RepeatProteinMask TEs % in genome | De novo Length (Bp) | De novo % in genome | Combined TEs Length (Bp) | Combined TEs % in genome |
| DNA | 40941255 | 1.97 | 1644537 | 0.08 | 1.68E+08 | 8.07 | 1.97E+08 | 9.47 |
| LINE | 20868427 | 1 | 6205696 | 0.3 | 52833806 | 2.54 | 64768467 | 3.12 |
| SINE | 117393 | 0.01 | 0 | 0 | 313613 | 0.02 | 430841 | 0.02 |
| LTR | 3.26E+08 | 15.68 | 3.41E+08 | 16.4 | 1.5E+09 | 72.04 | 1.53E+09 | 73.7 |
| Other | 32230 | 0 | 351 | 0 | 0 | 0 | 32581 | 0 |
| Unknown | 502156 | 0.02 | 0 | 0 | 56305996 | 2.71 | 56793143 | 2.73 |
| Total TE | 3.83E+08 | 18.42 | 3.49E+08 | 16.78 | 1.68E+09 | 81 | 1.73E+09 | 83.47 |

Supplementary Table 5 Statistical results of gene prediction

| Gene set | Number | Average gene length (bp) | Average CDS length (bp) | Average exon per gene | Average exon length (bp) | Average intron length (bp) |
| --- | --- | --- | --- | --- | --- | --- |
| denovo/AUGUSTUS | 49524 | 4880.43 | 1612.44 | 4.51 | 357.76 | 931.83 |
| denovo/Genscan | 44106 | 25626.66 | 1165.68 | 5.89 | 197.96 | 5003.88 |
| homo/D.carota | 65911 | 4322.12 | 949.43 | 3.46 | 274.36 | 1370.69 |
| homo/C.sativum | 73676 | 3827.33 | 803.68 | 3.28 | 245.03 | 1326.24 |
| homo/S.miltiorrhiza | 47550 | 3885.34 | 814.39 | 3.56 | 229 | 1201.35 |
| homo/A.thaliana | 43328 | 3679.21 | 857.85 | 3.78 | 226.84 | 1014.26 |
| homo/P.ginseng | 66345 | 4774.62 | 798.34 | 3.21 | 248.83 | 1800.52 |
| trans.orf/RNAseq | 12452 | 4747.16 | 1068.33 | 5.47 | 288.66 | 707.72 |
| MAKER | 44255 | 6374.66 | 1195.22 | 4.87 | 286.6 | 1286.81 |
| PASA | 42984 | 6498.51 | 1216.09 | 4.96 | 292.53 | 1266.55 |

Supplementary Table 6 Statistical results of gene function annotation

| Type | Number | Percent (%) |
| --- | --- | --- |
| Total | 42984 |  |
| InterPro | 31261 | 72.73 |
| GO | 32627 | 75.9 |
| KEGG_ALL | 41821 | 97.29 |
| KEGG_KO | 14844 | 34.53 |
| Swissprot | 29803 | 69.34 |
| TrEMBL | 42105 | 97.96 |
| NR | 42399 | 98.64 |
| Annotated | 42456 | 98.77 |
| Unannotated | 528 | 1.23 |
