# Supplementary figures and images for: Genomic, transcriptomic, and metabolomic analyses provide insights into the evolution and development of a medicinal plant Saposhnikovia divaricata (Apiaceae)
Source: Hortic Res. 2024 Apr 9;11(6):uhae105. doi: 10.1093/hr/uhae105 (PMC11179723; doi:10.1093/hr/uhae105)

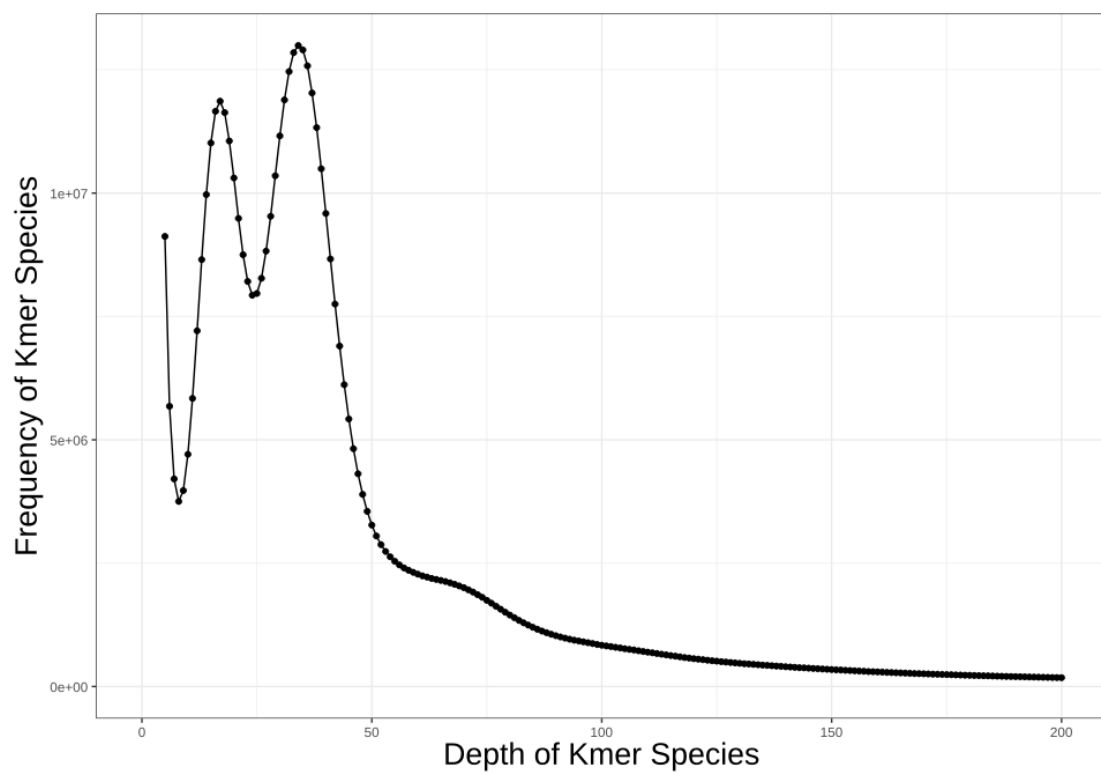

Supplement: Web_Material_uhae105 [file web_material_uhae105.zip › Supplementary Figure S1.pdf]

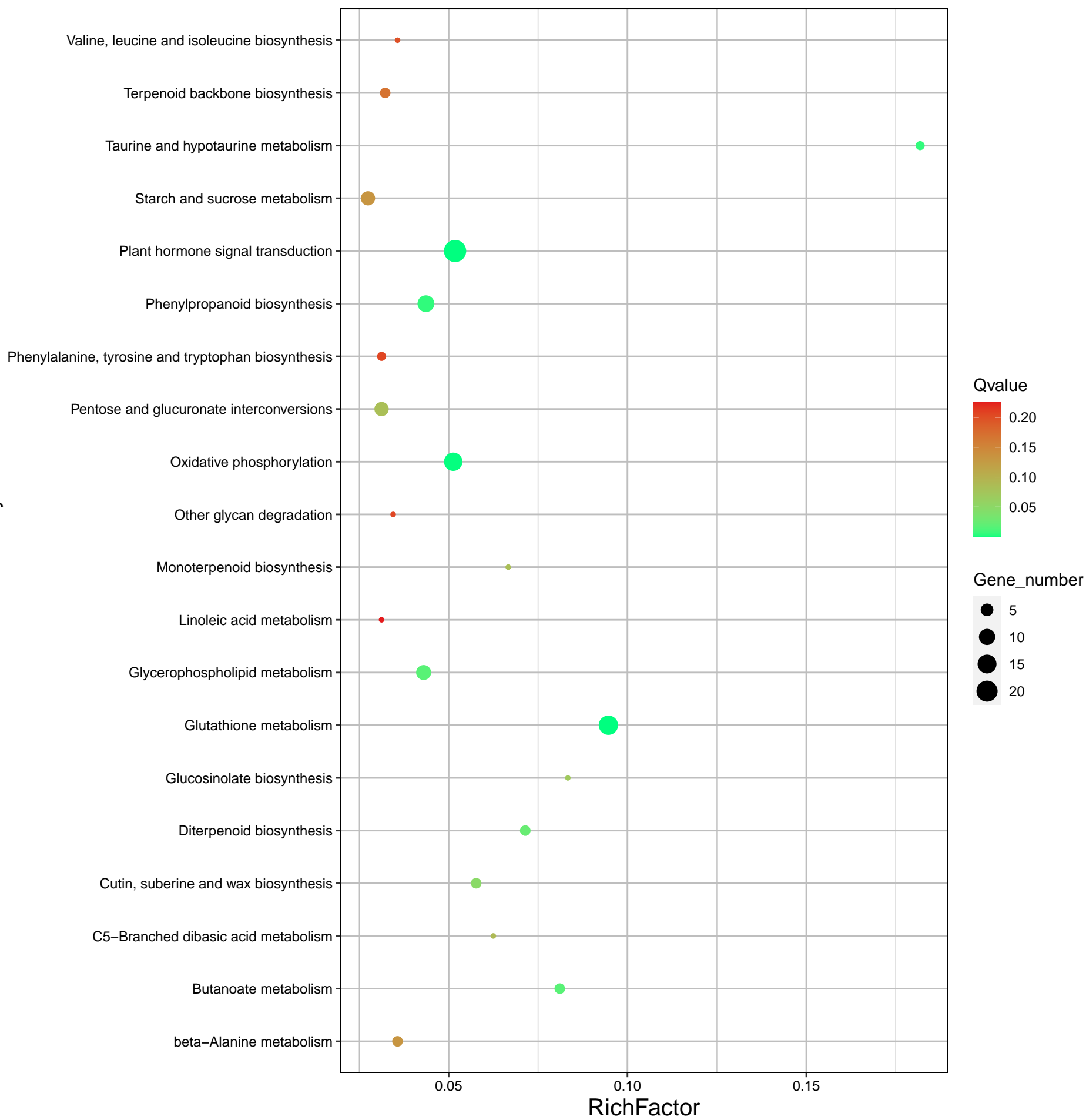

Supplement: Web_Material_uhae105 [file web_material_uhae105.zip › Supplementary Figure S2-contraction_0.05.plants.KEGG_enrichment.pdf]

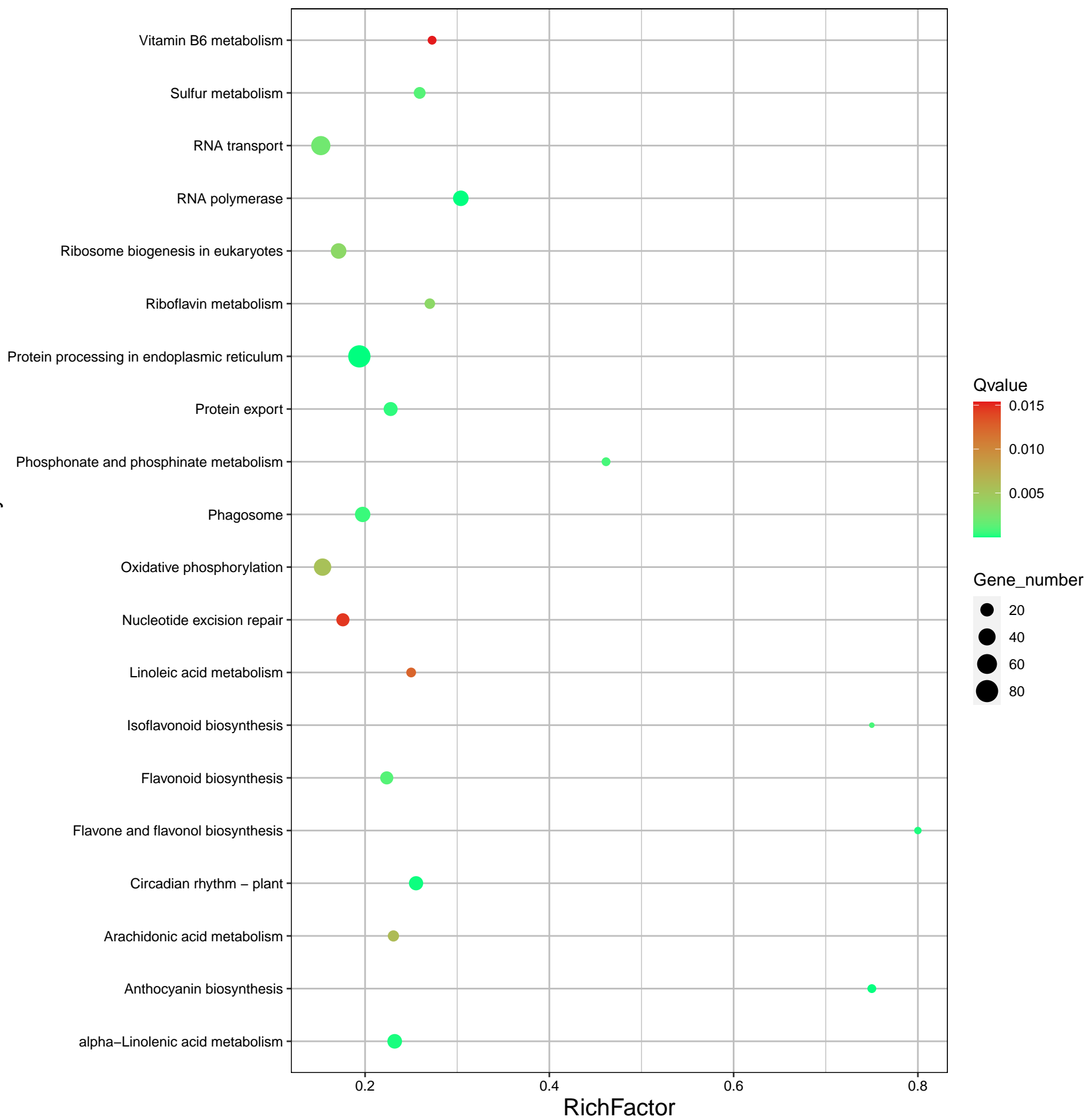

Supplement: Web_Material_uhae105 [file web_material_uhae105.zip › Supplementary Figure S3-expansion_0.05.plants.KEGG_enrichment.pdf]
